# Mental Health and Environmental Exposures: An Editorial

**DOI:** 10.3390/ijerph15102207

**Published:** 2018-10-10

**Authors:** Marco Helbich

**Affiliations:** Department of Human Geography and Spatial Planning, Faculty of Geosciences, Utrecht University, Princetonlaan 8a, 3584 CB Utrecht, The Netherlands; m.helbich@uu.nl; Tel.: +31-30-253-2017

**Keywords:** health geography, mental disorders, exposures, risk assessment, environments, environmental modelling

## 1. Introduction

It is well-documented that human mental health emerges from a complex interplay between genetic, psychological, lifestyle, and other factors. In addition, people are also exposed to numerous environments. These environmental exposures (e.g., green space, noise, air pollution, weather conditions, housing conditions) might trigger mental disorders or be protective factors, facilitating stress reduction, mental recovery, etc. [1,2]. In this special issue, “environmental exposure” is understood in the broadest sense, comprising natural (e.g., park, bodies of water, weather) [3], social (e.g., capital, cohesion) [4], and built environmental exposures (e.g., urbanicity, intersection density, land use mix) [5]. Although some environmental factors—e.g., air pollution and green space—have already received broad attention in scientific debates, others have received very little, resulting in a tentative and partly inconclusive understanding of the environment–mental health relationship.

Mental illness contributes significantly to the global burden of mental disorders (i.e., 13% disability adjusted life-years lost) [6]. It is therefore important to grasp how and to what extent environmental exposures affect mental health outcomes. In the past year, 20% of all adults worldwide suffered from a mental disorder. Mental disorders have a lifetime prevalence of two out of seven adults and will continue to remain a leading cause of disease burden [7]. Such disorders have devastating consequences for people’s quality of life and represent striking challenges for health systems as a whole. Thus, the reduction of mental disorders is a health priority in both developed and developing countries.

The geographic context of individuals is a central construct in assessing the contribution of environmental exposures to people’s mental health [2]. While residential neighborhoods are frequently thought to represent an environmental context, this approach is increasingly critiqued because it assumes that people are immobile and exposed only to their residential neighborhoods. As this seems to be too restrictive an assumption, mobility-based environmental exposure assessments in mental health research have been put forward as methods that represent exposures more accurately. Such approaches highlight the importance of exposures that people experience throughout the day and over their lifetime [2].

## 2. Objective of the Special Issue

The collection of international case studies presented in this special issue contribute to a better understanding of which environmental exposures affect mental health outcomes, as well as how and to what extent they do so. These case studies provide novel insights into the interaction between mental health and the environment (e.g., green space and natural disasters). To present state-of-the-art methods and to further stimulate lively discussions on this topic, scholars were invited to submit original research, methodological papers, reviews, and meta-analyses related to the entire spectrum of mental disorders (e.g., depression, schizophrenia). This special issue also features papers documenting how scientific findings are translated into prevention strategies, health policies, and clinical practices.

## 3. The Papers

By the time of the submission deadline (i.e., the end of August 2018), a total of 10 manuscripts were accepted after a single-blind review process by at least two international experts using the journal-specific review guidelines. As usual, the scientific quality of the research and its methodological soundness had a crucial influence on whether a manuscript was accepted. If major revisions were requested by the reviewers, or needed to guarantee high scientific quality, a second review of the revised manuscript was conducted by at least one of the original reviewers or an alternative reviewer. If a review called for only minor revisions, a second review was not conducted. Instead, the guest editor decided whether the revised manuscript was fit for publication.

The first study by Nichani and colleagues [8] used cohort data from New Zealand to investigate whether the distance of an individual’s residential location to the nearest green space affects depression risk during pregnancy. No evidence was found to support the hypothesis that maternal exposure to green space lowers the risk of antenatal depression. Similarly, after investigating patients in Utrecht, Netherlands, Boers et al. [9] found no significant associations between hospital admissions for psychotic disorders and exposure to green and blue space. The experience of natural disasters such as hurricanes, however, can have long-lasting effects on people’s mental health outcomes. A two-paper series by Schwartz and colleagues addressed this by studying the impact of hurricanes Sandy [10] and Harvey [11] on numerous mental health symptoms using New York City and Long Island residents’ data. Longitudinal analysis provided evidence that, for example, personal and property damage caused by hurricanes evoked symptoms of post-traumatic stress disorders but, in the case of Hurricane Sandy, not anxiety and depression symptoms. Natural disasters are not the only occurrences to have adverse effects on mental health outcome. Daily weather conditions are increasingly reported to influence suicide mortality [12]. No evidence for associations between suicide risk, daily temperature, and rainfall was found by Fernández-Niño et al. [13] for Columbian cities. Scientific evidence is mounting that mental health, in general, and suicide mortality, in particular, are related not only to personal characteristics and life events but also to environmental exposures other than weather conditions [14,15]. Two examples are reported in this special issue. Firstly, in a nationwide ecological study of the USA, Ha and Tu [16] showed that altitude is positively related to suicide, though this association seems to vary spatially. Secondly, Wang and colleagues [17] found that air pollution in China adversely affected depression symptoms, while neighborhood social capital seems to be a protective factor. Other than the social environment on a neighborhood level, close family also plays a crucial role in the development of mental disorders. For example, Guček and Selič [18] showed that exposure to intimate partner violence was a significant risk factor for the prevalence of depression, as were such life events as divorce. Xiao et al. [19] showed by means of structural equation models that housing conditions in Shanghai, China indirectly influenced migrants’ mental health, whereas locals were directly affected. From a spatial planning point of view, the provision of environments supporting people’s physical activity is central, as walkable areas reduce the risk of experiencing mental disorders. The study by Mayne et al. [20] addressed whether psychological distress is correlated with the walkability of the built environment at the zip code level in Sydney, Australia. Based on the absence of an association, the authors advised that health policies should focus on the personal level.

In conclusion, some of the papers in this special issue support the notion that environments can affect, in one way or the other, people’s mental health. Although these studies advance our understanding of environment–health relations, there are several gaps in the context of the aforementioned contributions and in the literature on environmental health as a whole. For example, a key challenge for future research is how environmental exposures are assessed. It is traditionally assumed that residential location is the sole exposure source. However, the fragmentation of people’s daily lives across numerous activity locations, as well as their residential mobility over the course of their lives, makes this approach questionable and calls for more comprehensive and dynamic exposure assessments [2]. Future research is advised to make the traversed environment central, as it might contribute to the onset of a mental disorder, and to integrate not only exposures at the actual place of residence but also those around past residential locations, as they may contribute to mental health disorders later in life.
